# Synthetic anion channels: achieving precise mimicry of the ion permeation pathway of CFTR in an artificial system†

**DOI:** 10.1039/d4sc06893a

**Published:** 2024-11-25

**Authors:** Linlin Mao, Shuaimin Hou, Linlin Shi, Jingjing Guo, Bo Zhu, Yonghui Sun, Junbiao Chang, Pengyang Xin

**Affiliations:** a State Key Laboratory of Antiviral Drugs, Pingyuan Laboratory, NMPA Key Laboratory for Research and Evaluation of Innovative Drug, School of Chemistry and Chemical Engineering, Henan Normal University 46 Jianshe Road Xinxiang 453007 Henan China syonghui1994@163.com pyxin27@163.com +86 373 3328652; b Centre in Artificial Intelligence Driven Drug Discovery, Faculty of Applied Sciences, Macao Polytechnic University Macao 999078 China jguo@mpu.edu.mo

## Abstract

CFTR (Cystic Fibrosis Transmembrane Conductance Regulator), a naturally occurring anion channel essential for numerous biological processes, possesses a positively charged ion conduction pathway within its transmembrane domain, which serves as the core module for promoting the movement of anions across cell membranes. In this study, we developed novel artificial anion channels by rebuilding the positively charged ion permeation pathway of the CFTR in artificial systems. These synthetic molecules can be efficiently inserted into lipid bilayers to form artificial ion channels, which exhibit a preference for anions during the transmembrane transport process. More importantly, the positively charged amino acid residues located in the ion permeation pathway of these artificial channels can promote the transmembrane transport of anions through electrostatic interactions, which is consistent with the mechanism of anion transmembrane transport achieved by CFTR.

## Abstract 2

The distribution pattern of positively charged amino acid residues within the ion permeation pathway of CFTR (Cystic Fibrosis Transmembrane Conductance Regulator) has been replicated in an artificial system.

## Graphical abstract

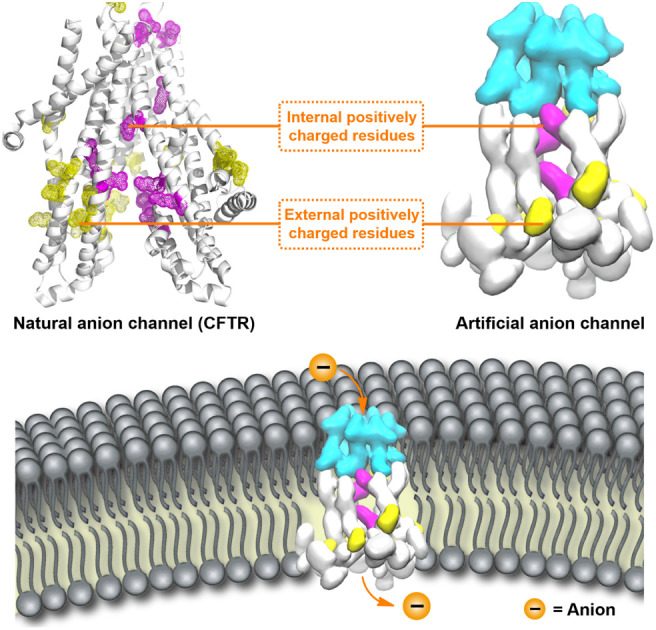

## Introduction

**Fig. 1:**
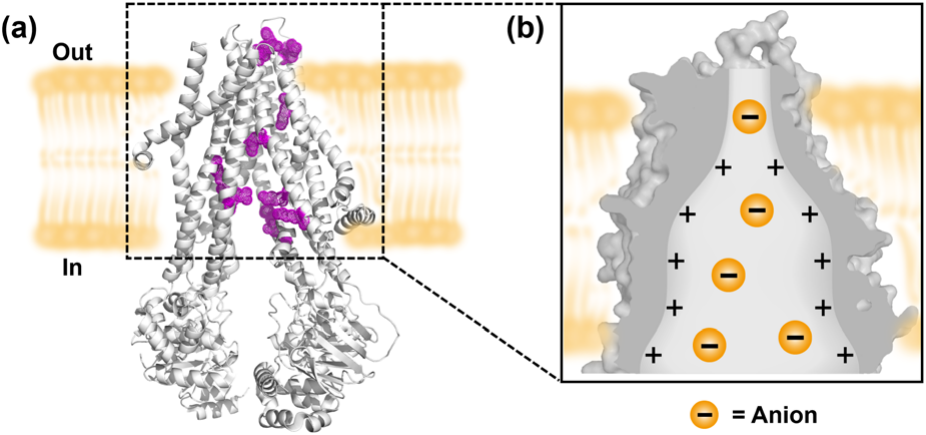
(a) Structural features of CFTR, with positively charged residues located within the ion transport pathway highlighted in purple. (b) Schematic representation of the inverted funnel-shaped ion permeation pathway.

The transmembrane transport of anions is crucial for many physiological processes, such as regulating cellular pH and maintaining cell volume and osmotic balance.^1^ Normally, the anion homeostasis of cells is regulated by native proteins, which mediate the anion transport through the “carrier” or “channel” mechanism.^2^ CFTR (Cystic Fibrosis Transmembrane Conductance Regulator) is one of the most important anion channels expressed in many different cell types (Fig. 1a).^3^ Unlike general integral membrane proteins, which predominantly contain hydrophobic residues to form transmembrane segments,^4^ CFTR possesses unique transmembrane domains (TMDs) that incorporate a significant number of arginines and lysines.^5^ Furthermore, differently from the traditionally accepted ‘positive-inside’ rule, some positively charged amino acid residues reside within the ion permeation pathway formed by the TMDs of CFTR. Therefore, the inverted funnel-shaped ion permeation pathway is highly positive and lined with polar and charged residues, which provide a high affinity for anions and offer an electrostatic barrier to the entry of cations into the channel pore (Fig. 1b).^6^ The dysfunction of CFTR will lead to dysregulation of anion transport, which is associated with a number of diseases known as “channelopathies”, such as cystic fibrosis (CF).^7^

On account of the fact that natural anion channels play an important role in various physiological processes, attempts to use artificial systems to mimic the natural anion channels have emerged in recent years.^8^ Various structural elements such as rigid rods,^9^ α-aminoxy acids,^10^ macrocycles,^11^ isophthalamides,^12^ diols,^13^ peptide derivatives,^14^ and other structures,^15^ are used as scaffolds to construct anion channels. These elegant alternatives could serve as simplified models to understand the transport mechanism of natural anion channels and may find potential applications in channel-related drug discovery.^16^ However, the topological structure and anion recognition mechanisms in most of the reported examples are quite different from those in natural anion channels, which may bring uncertainty to further biological applications. Hence, there is still plenty of scope for further exploration of new synthetic anion channels with more structural features and transport behaviors similar to those of native anion channels. Herein, we designed two synthetic anion channels by rebuilding the positively charged ion permeation pathway of CFTR in artificial systems. These anion channels not only possess similar core modules to their natural prototypes but also exhibit comparable transport behavior, which have the potential to serve as biomimetic alternatives to CFTR.

**Fig. 2:**
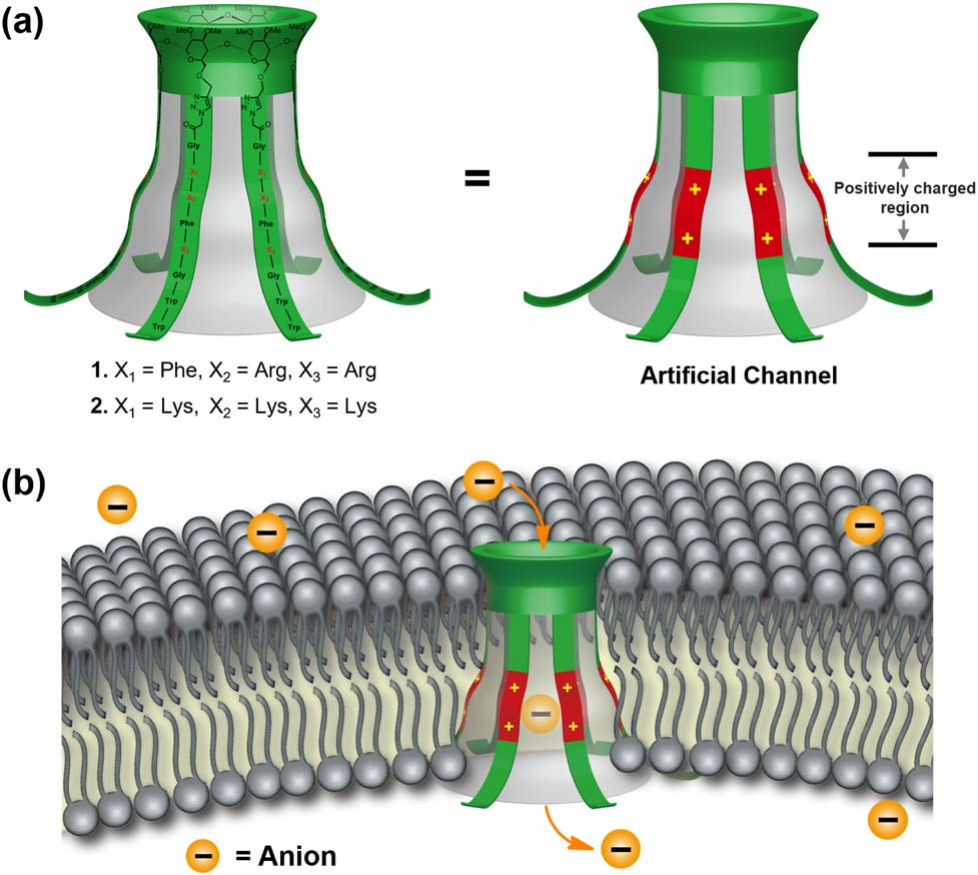
(a) Molecular design of the artificial channels 1–2. (b) Schematic representation of anion channels hypothesized to form by 1–2 in a lipid bilayer.

α-Cyclodextrin (α-CD) possesses a bucket-shaped cavity with an inner diameter of ≈5.0 Å, which is the ideal scaffold for constructing synthetic channels.^17^ By attaching multiple positively charged peptide chains to the primary hydroxy groups of α-CD, we could rebuild the positively charged and inverted funnel-shaped ion permeation pathway of CFTR in an artificial system.^6^ To enhance the membrane-incorporation ability, multiple tryptophan (Trp) residues were introduced at the ends of the tubular molecules 1 and 2 (Fig. 2a).^18^ We envisioned that the design above allows us to rebuild the core modules of CFTR in an artificial system, mimicking the structural features and transport behavior of native anion channels (Fig. 2b).

## Results and discussion

**Fig. 3:**
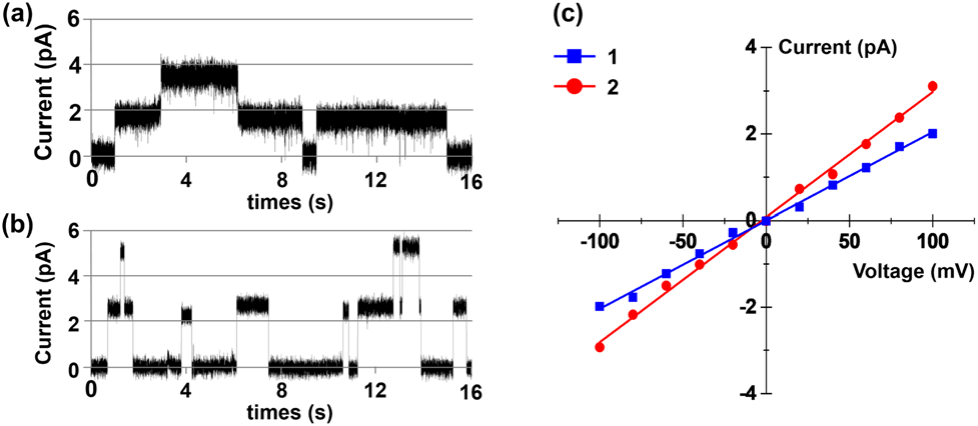
(a) Current traces through a diPhyPC lipid bilayer in 1.0 M KCl at a potential of +80 mV in the presence of (a) 1 and (b) 2. (c) *I*–*V* plots of 1 and 2 in the planar lipid bilayer in asymmetric electrolytes.

We prepared tubular molecules 1 and 2 from the click reaction of the corresponding azido-peptides and per-6-alkynyl-α-cyclodextrin, and the structures of these molecules were characterized by NMR spectroscopy and MS (see Section 2 in the ESI†). To demonstrate the transport mechanism and membrane activity of 1 and 2, bilayer lipid membrane (BLM) electrophysiology measurements were performed (see Section 3 in the ESI†). For these experiments, two chambers containing KCl solution (1.0 M) were separated by a planar lipid bilayer composed of diphytanoylphosphatidylcholine (diPhyPC). A solution of 1 and 2 in DMSO was added into the *cis* chamber to reach a final concentration of 0.2 μM. After the addition of the compounds, regular square-like and long-lived single channel currents were observed upon applying a voltage of +80 mV across the membrane (Fig. 3a and b). These observations provide strong evidence that the tubular molecules could incorporate into the lipid bilayer and form transmembrane channels.^19^ The current–voltage (*I*–*V*) plots of 1 and 2 were obtained from BLM electrophysiology measurements at different voltages, which displayed a linear *I*–*V* relationship in the range of −100 to +100 mV (Fig. 3c). By using the resulting *I*–*V* plots, the corresponding conductance (*γ*) values were calculated to be 20.4 ± 0.4 (1) and 28.9 ± 0.5 pS (2). The γ value of channel 2 is higher than that of 1 and indicates that 2 is more effective in transporting ions.

**Fig. 4:**
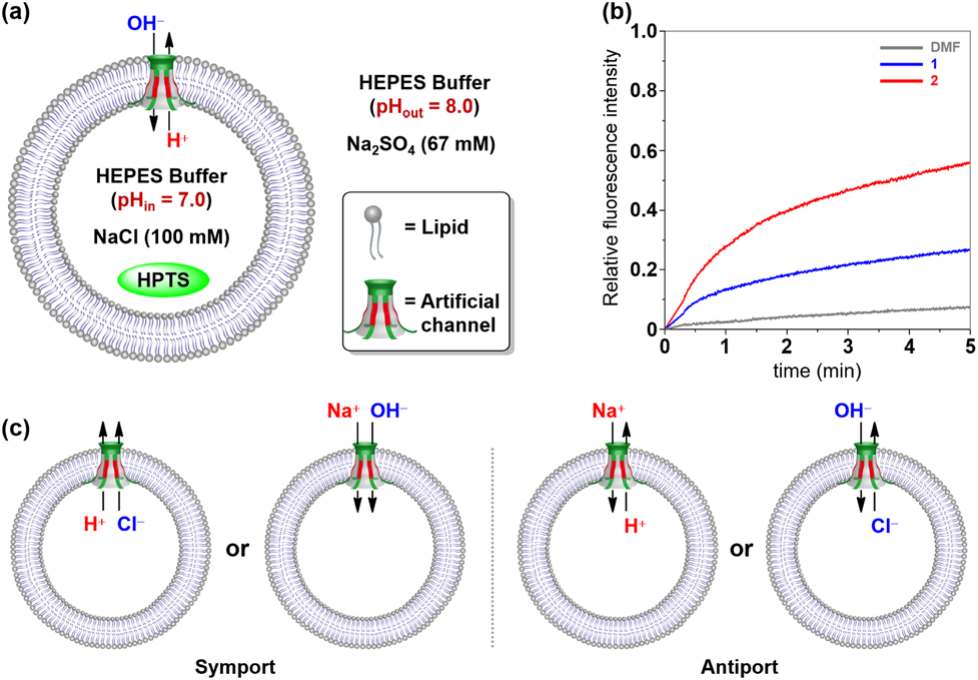
(a) Schematic representation of the HPTS assay. (b) Changes in the fluorescence intensity of HPTS (*λ*_ex_ = 460 nm and *λ*_em_ = 510 nm) in vesicles with time after the addition of 1 and 2 (*x* = 1.25%, molar ratio relative to the lipid, represented by *x*). (c) Schematic representation of the possible ion-transport mechanism and species.

Having established that tubular molecules 1 and 2 mediate the transmembrane transport of ions through a channel mechanism, fluorescence assays based on vesicles were further used to probe the transport species of these channels.^20^ Firstly, a suspension of LUVs (large unilamellar vesicles) composed of EYPC (egg yolk phosphatidylcholine) entrapping the pH-sensitive dye HPTS (8-hydroxypyrene-1,3,6-trisulfonate) was first prepared (10 mM HEPES, 100 mM NaCl, and pH = 7.0). Then a pH gradient across the membranes was introduced by the addition of the LUV solution to a buffer (10 mM HEPES, 67 mM Na_2_SO_4_ and pH = 8.0) (Fig. 4a). After the addition of channels 1 and 2 to the vesicle suspensions, the fluorescence intensity of HPTS increased to 27% and 56%, respectively (Fig. 4b). These results indicate that these tubular molecules could mediate the H^+^ efflux or OH^−^ influx, which could be due to the charge balance through symport (H^+^/Cl^−^ and Na^+^/OH^−^) or antiport (H^+^/Na^+^ and Cl^−^/OH^−^) mechanisms (Fig. 4c).

**Fig. 5:**
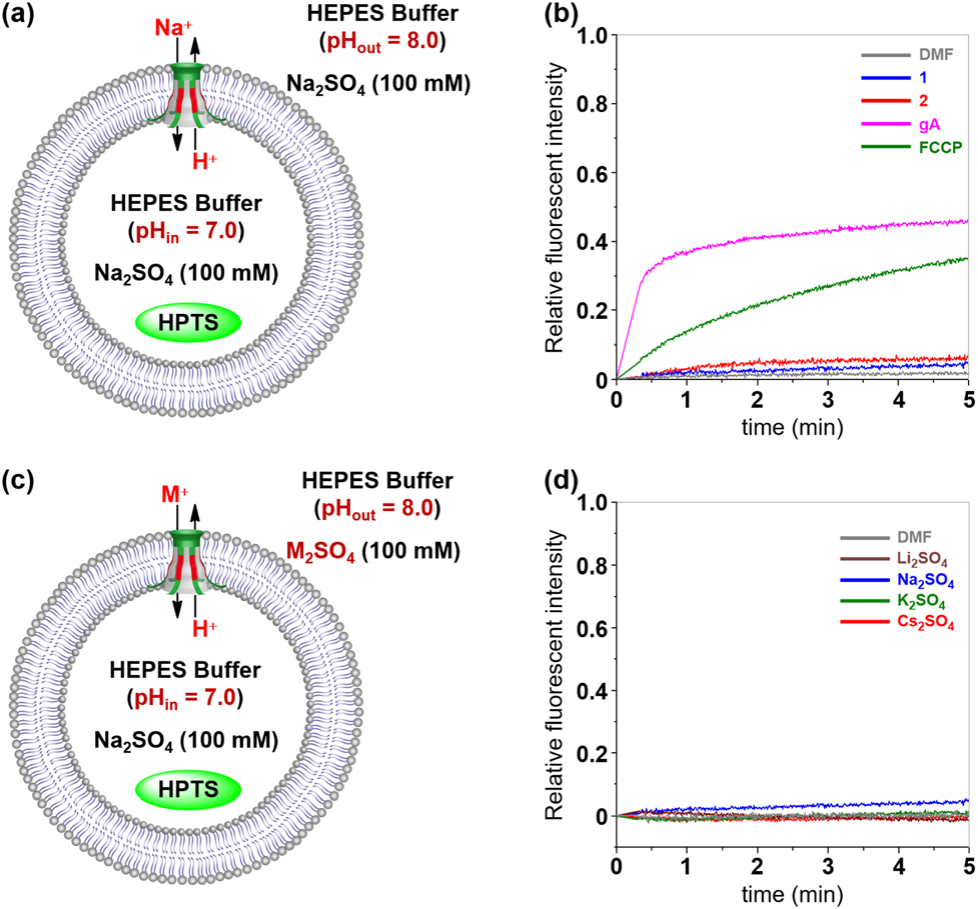
(a) LUV-based HPTS assay for the transport species study. (b) Changes in the fluorescence intensity of HPTS after the addition of 1, 2, gA and FCCP. (c) HPTS assay with various external metal ions (M^+^ = Li^+^, Na^+^, K^+^ and Cs^+^). (d) Changes in the fluorescence intensity during the cation transport activity test of 1.

To further confirm the transport species of 1 and 2, two sets of HPTS assays were performed.^15*m*^ Firstly, the tubular molecules, gramicidin A (gA, cation channel) and carbonyl cyanide 4-(trifluoro methoxy)phenylhydrazone (FCCP, proton transporter), were evaluated using LUVs containing HPTS, HEPES (10 mM) and Na_2_SO_4_ (100 mM) at pH 7.0, which were subsequently exposed to a pH gradient by adding to a buffer solution (10 mM HEPES, 100 mM Na_2_SO_4_, and pH = 8.0) (Fig. 5a). With the addition of gA and FCCP, the fluorescence intensity of HPTS increased to 45% and 35%, respectively (Fig. 5b). These results demonstrated that gA and FCCP possess substantial transport activity towards transport of H^+^ and Na^+^. Under identical conditions, artificial channels 1 and 2 were almost irrelevant to the fluorescence intensity of HPTS. These results reveal that neither H^+^/Na^+^ antiport nor Na^+^/OH^−^ symport is a likely mechanism during the ion transport process. The above observation was further confirmed by varying the external metal ions (M^+^ = Li^+^, Na^+^, K^+^ and Cs^+^) (Fig. 5c). As seen in Fig. 5d and S18 (see the ESI†), both channels 1 and 2 exhibit very low transport activity for the tested metal ions. The above studies clearly indicated that anions rather than cations mainly participated in the ion transport process.

**Fig. 6:**
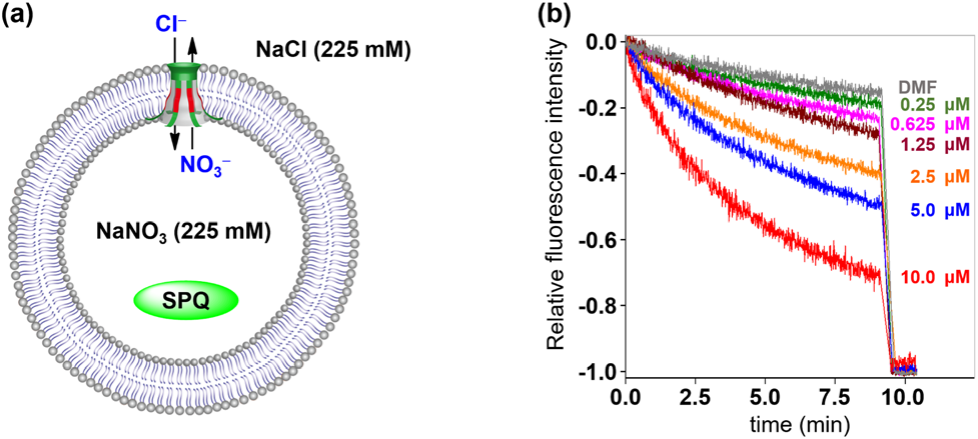
(a) LUV-based SPQ assay for the transport species study. (b) Changes in the fluorescence intensity of SPQ after the addition of 1.

Then, fluorescence experiments were carried out in the presence of a chloride-sensitive SPQ (6-methoxy-*N*-(3-sulfopropyl)quinolinium) dye to evaluate the anion transmembrane transport abilities of channels 1 and 2 (Fig. 6a).^14*b*^ Briefly, The LUVs loaded with SPQ (30 μL, 10 mM in 225 mM NaNO_3_) were exposed to a chloride gradient by adding to a NaCl solution (2.0 mL, 225 mM). As shown in Fig. 6b and S21 (see the ESI†), the fluorescence intensity of SPQ decreased in a concentration-dependent manner upon addition of channels 1 and 2. This observation clearly indicated that Cl^−^ is one of the main species, which is involved in the transmembrane transport process of these artificial channels.

**Fig. 7:**
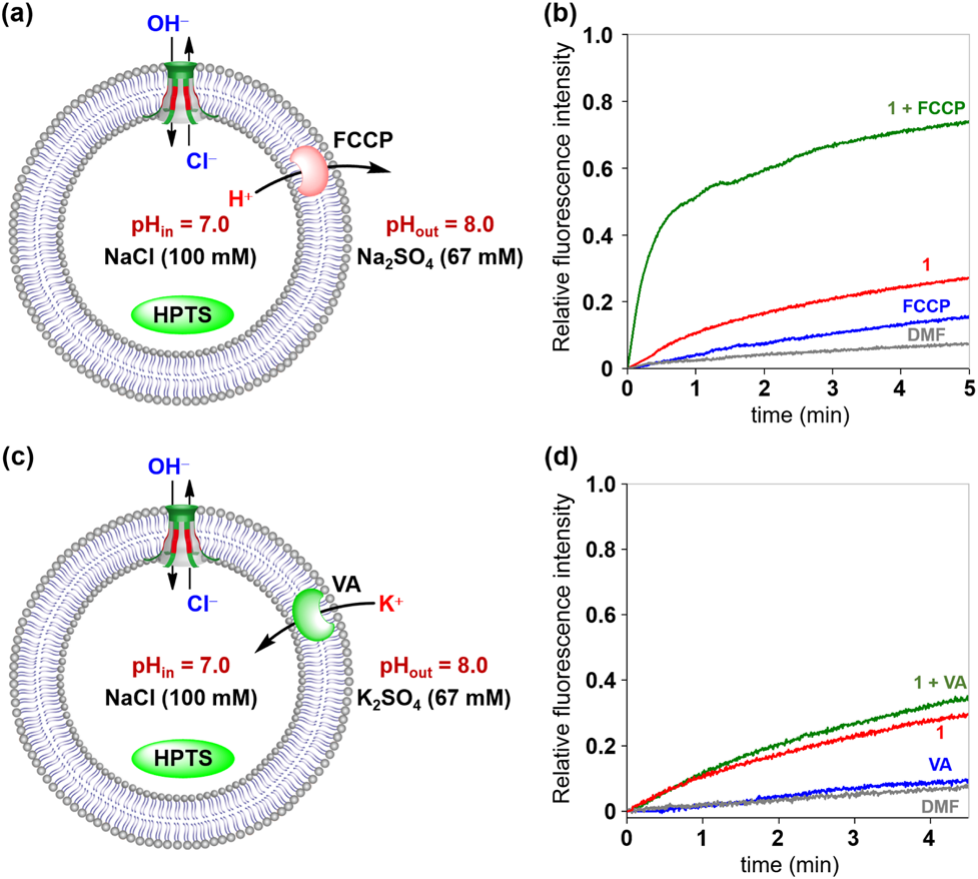
(a) Schematic representation of the FCCP-HPTS assay. (b) Changes in the fluorescence intensity of HPTS after the addition of 1 and FCCP. (c) Schematic representation of the VA-HPTS assay. (d) Changes in the fluorescence intensity of HPTS after the addition of 1 and VA.

The above two fluorescence assays presented in Fig. 5 and 6 have clearly indicated that chloride ions can be transported across the membrane *via* Cl^−^/OH^−^ antiport or H^+^/Cl^−^ symport mechanisms. To gain a deeper insight into the transport mechanism of these channels, the commercial H^+^ transporter FCCP and K^+^ carrier VA (valinomycin) were applied in the HPTS assays, respectively (Fig. 7).^21^ In the FCCP-HPTS assay, the transport efficiency of channel 1 and FCCP was 27.3% and 15.8%, respectively. When channel 1 and FCCP were co-injected into the LUV suspension, the fluorescence intensity of HPTS significantly increased to 73.9% (Fig. 7a and b). These results demonstrated that the co-injected FCCP could increase the transport rate of H^+^, which further indicates that the H^+^ or OH^−^ transport efficiency of channel 1 is much slower than that of Cl^−^. Then, the VA-HPTS assay was performed to compare the transport rates of H^+^, OH^−^, and Cl^−^ (Fig. 7c and d). In this assay, the presence of VA will selectively mediate the K^+^ transport from outside to inside because of the concentration gradient and further results in the efflux of H^+^ or the influx of OH^−^ through the anion channel to sustain the overall charge balance. Simultaneously, the Cl^−^ present within the LUVs will also undergo efflux through the anion channel, driven by the concentration gradient. Once the flow rate of H^+^ or OH^−^ is higher than that of Cl^−^, a significant increase in the fluorescence intensity of HPTS will be observed. However, compared with the individual addition of channel 1 (29.4%), the co-injection of channel 1 and VA did not improve the fluorescence intensity of HPTS significantly (35.7%). These observations suggest that Cl^−^ possesses a higher flow rate than H^+^ or OH^−^ during the transport process. The above two assays further confirmed that the H^+^/Cl^−^ symport and Cl^−^/OH^−^ antiport are the main transport mechanisms to sustain the overall charge balance during the transport process, and Cl^−^ is the preferred species among the above three transported ions (H^+^, Cl^−^ and OH^−^).

**Fig. 8:**
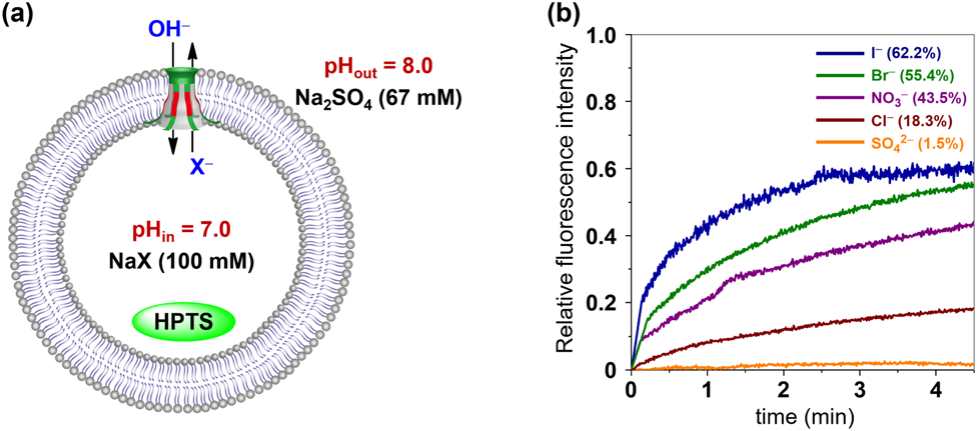
(a) HPTS assay with various internal anionic ions (X^−^ = Cl^−^, Br^−^, I^−^, NO_3_^−^, SO_4_^2−^). (b) Changes in the fluorescence intensity during the anion transport activity test of 1.

The anion selectivities of 1 and 2 were also investigated by using the HPTS assay, as illustrated in Fig. 8a.^15*i*^ Changes in the fluorescence of HPTS-labeled LUVs were determined by varying the anionic sodium salts within the LUVs (NaX, X^−^ = Cl^−^, Br^−^, I^−^, NO_3_^−^ and SO_4_^2−^). The resulting fluorescence intensity traces were normalized and baseline-corrected to enable a more accurate comparison of the transport activity of these synthetic channels towards various anions. As shown in Fig. 8b and S23,† despite variations in the amino acid residues lining their ion permeation pathways, channel molecules 1 and 2 demonstrate very similar ion selectivities, both conforming to the sequence I^−^ > Br^−^ > NO_3_^−^ > Cl^−^ > SO_4_^2−^.

**Fig. 9:**
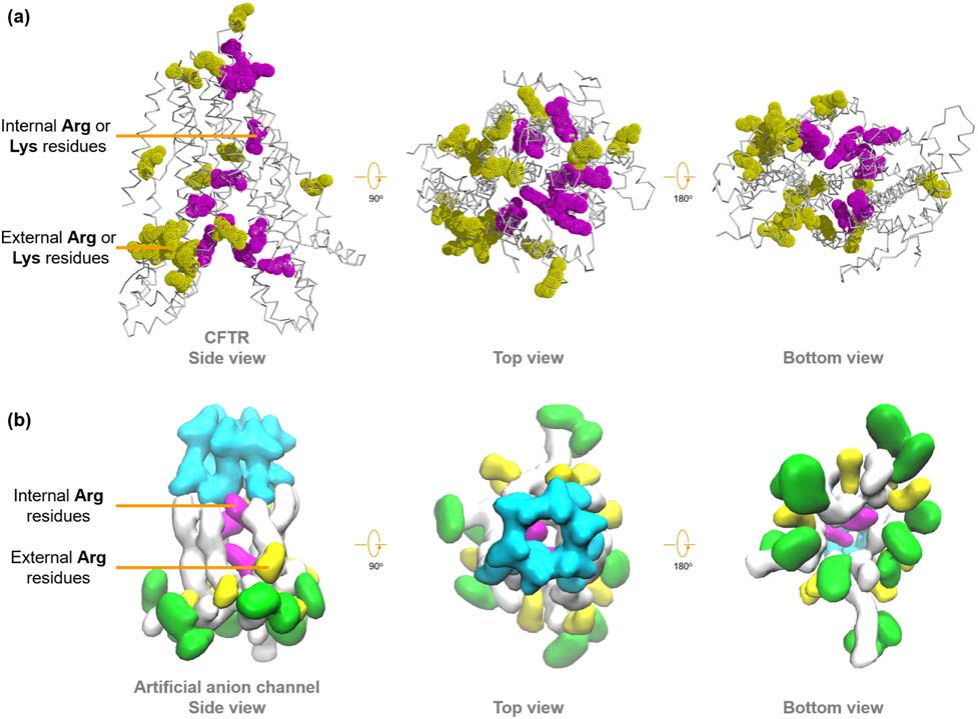
The distribution diagrams of positively charged amino acid residues in (a) the membrane-spanning domain of CFTR and (b) channel 1. The residues located within the ion transport pathway are highlighted in purple, while those far from the ion transport pathway are highlighted in yellow. The Trp residues in channel 1 are marked in green.

Recent studies have categorized the positively charged amino acid residues in the membrane-spanning domain of CFTR into two distinct groups. The first group resides within the inverted funnel-shaped ion permeation pathway of CFTR. The side chains of these residues do not engage in specific interactions with other parts of the protein, allowing them to contribute to a positively charged microenvironment to the ion permeation pathway while also facilitating anion binding (Fig. 9a, highlighted in purple). In contrast, the second group of positively charged residues is positioned away from the ion permeation pathway, predominantly on the external surface of CFTR, and does not impact the transmembrane transport of anions (highlighted in yellow).^4–6^ Similar to CFTR, our artificial anion channels also possess multiple positively charged amino acid residues. To investigate whether the distribution patterns of positively charged amino acid residues in these artificial anion channels mirror those found in their natural prototypes, further molecular dynamics simulations were performed (see Section 9 in the ESI†). As depicted in Fig. 9b, α-CD provides a stable scaffold for channel 1 and assembles with multiple positively charged peptide chains to form a hollow tubular structure, thereby offering an ion conduction pathway for transmembrane anion transport. Most Arg residues (9 out of 12) in channel 1 are located on the external surface of the channel molecule, while notably, three Arg residues are discernibly positioned within the ion permeation pathway of this artificial channel. This observation suggests that the positively charged amino acid residues in our artificial anion channel exhibit a similar distribution pattern to that of CFTR.

**Fig. 10:**
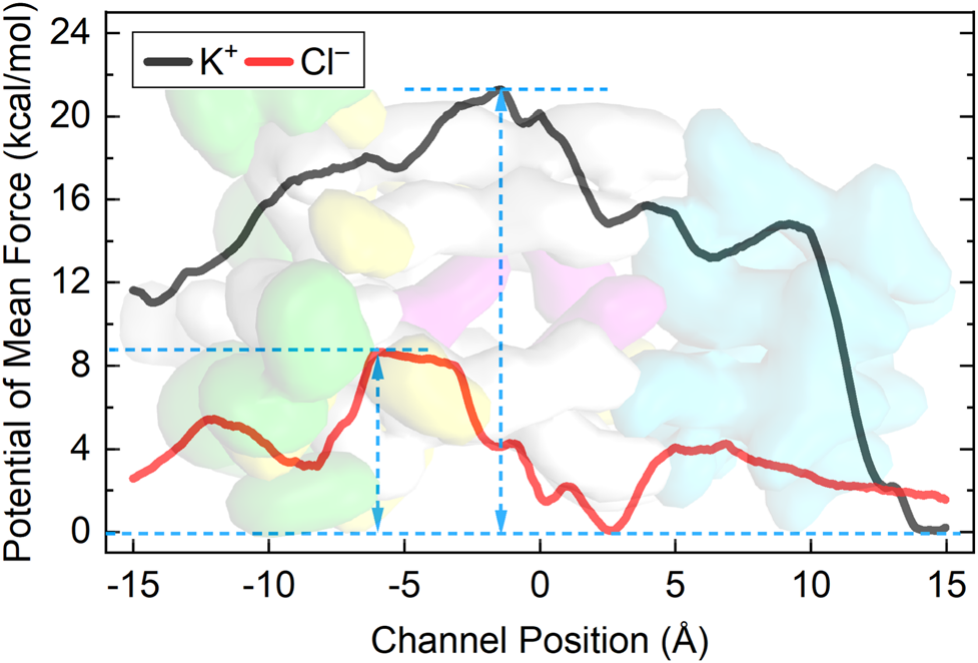
The distribution diagrams of positively charged amino acid residues in (a) the membrane-spanning domain of CFTR and (b) channel 1. The residues located within the ion transport pathway are highlighted in purple, while those far from the ion transport pathway are highlighted in yellow. The Trp residues in channel 1 are marked in green.

To further explore the relationship between the distribution of positively charged amino acid residues and ion selectivity in these artificial anion channels, the energy barriers along this pathway were determined using the potential of mean force (PMF) with adaptive biasing forces to understand how the channel discriminates between anions and cations (see Section 9 in the ESI†). As depicted in Fig. 10, the energy barrier for K^+^ to enter and pass through the channel is much higher than that for Cl^−^. Intriguingly, Cl^−^ ions display local energy minima as they traverse the Arg-rich segment within the ion permeation pathway, in contrast to K^+^ ions, which encounter local energy maxima in the same region. This computational result was confirmed by the *P*_Cl−_/*P*_K+_ values obtained from planar bilayer conductance measurements, which were calculated to be 11.5 (1) and 1.5 (2) (Fig. S17, ESI†), clearly indicating that these channels possess a higher preference for transporting Cl^−^ during transmembrane transportation. The above observations suggest that the electrostatic interaction between transported ions and positively charged amino acid residues in the ion permeation pathway plays a crucial role in determining the preference of these channel molecules for anion transportation, resembling the ion selectivity mechanism observed in CFTR.

## Conclusions

In conclusion, artificial anion channels were constructed by rebuilding the positively charged ion permeation pathway of the natural anion channel protein CFTR in artificial systems. Electrophysiological experiments and LUV-based fluorescence experiments demonstrated that these synthetic molecules can be efficiently inserted into lipid bilayers to form artificial ion channels, which exhibit a preference for anions during the transmembrane transport process. More importantly, the positively charged amino acid residues located in the ion permeation pathway can promote the transmembrane transport of anions through electrostatic interaction, which is consistent with the mechanism of anion transmembrane transport achieved by CFTR. The findings presented herein provide biomimetic alternatives that exhibit structural characteristics and transport behaviors similar to those observed in native anion channels, thereby holding promising potential for applications in molecular devices and pharmaceutical technologies.

## Data availability

The data supporting this article have been included as part of the ESI.†

## Author contributions

P. X. and Y. S. conceived the idea and designed the research. L. M. and S. H. synthesized molecules together and performed the ionophoric experiments, ion transport mechanism studies, and chloride transport activity and anion transport activity studies. L. S. performed the cation transport experiments. J. G. carried out the theoretical calculations. P. X. and Y. S. wrote the manuscript of this work. B. Z. and J. C. provided constructive suggestions for results and helped revise the paper. All authors participated in the discussion.

## Conflicts of interest

The authors declare no competing interest.

## Supplementary Material

SC-016-D4SC06893A-s001

## References

[cit1] (a) Hille B. , Ionic Channels of Excitable Membranes, Sinauer Associates, Sunderland, 3rd edn, 2001

[cit2] Eisenman G., Horn R. (1983). J. Membr. Biol..

[cit3] Csanády L., Vergani P., Gadsby D. C. (2019). Physiol. Rev..

[cit4] Heijne G. V. (1992). J. Mol. Biol..

[cit5] Zhang Z., Chen J. (2016). Cell..

[cit6] Liu F., Zhang Z., Csanády L., Gadsby D. C., Chen J. (2017). Cell..

[cit7] Sheppard D. N., Rich D. P., Ostedgaard L. S., Gregory R. J., Smith A. E., Welsh M. J. (1993). Nature.

[cit8] Davis A. P., Sheppard D. N., Smith B. D. (2007). Chem. Soc. Rev..

[cit9] Gorteau V., Bollot G., Mareda J., Perez-Velasco A., Matile S. (2006). J. Am. Chem. Soc..

[cit10] Li X., Shen B., Yao X.-Q., Yang D. (2007). J. Am. Chem. Soc..

[cit11] Sidorov V., Kotch F. W., Abdrakhmanova G., Mizani R., Fettinger J. C., Davis J. T. (2002). J. Am. Chem. Soc..

[cit12] Sakai N., Houdebert D., Matile S. (2003). Chem.–Eur. J..

[cit13] Saha T., Dasari S., Tewari D., Prathap A., Sureshan K. M., Bera A. K., Mukherjee A., Talukdar P. (2014). J. Am. Chem. Soc..

[cit14] Schlesinger P. H., Ferdani R., Liu J., Pajewska J., Pajewski R., Saito M., Shabany H., Gokel G. W. (2002). J. Am. Chem. Soc..

[cit15] Jiang C., Lee E. R., Lane M. B., Xiao Y.-F., Harris D. J., Cheng S. H. (2001). Am. J. Physiol. Lung Cell Mol. Physiol..

[cit16] Davis J. T., Okunola O., Quesada R. (2010). Chem. Soc. Rev..

[cit17] Szejtli J. (1998). Chem. Rev..

[cit18] Fonseca V., Daumas P., Ranjalahy-Rasoloarijao L., Heitz F., Lazaro R., Trudelle Y., Andersen O. S. (1992). Biochemistry.

[cit19] (a) Ashley R. H. , Ion Channels: A Practical Approach, Oxford University Press, Oxford 1995

[cit20] Jeon Y. J., Kim H., Jon S., Selvapalam N., Oh D. H., Seo I., Park C.-S., Jung S. R., Koh D.-S., Kim K. (2004). J. Am. Chem. Soc..

[cit21] Rose L., Jenkins A. T. A. (2007). Bioelectrochemistry.

