# Supplementary material for: Synthetic anion channels: achieving precise mimicry of the ion permeation pathway of CFTR in an artificial system
Source: Chem Sci. 2024 Nov 25;16(1):371–7. doi: 10.1039/d4sc06893a (PMC11605520; doi:10.1039/d4sc06893a)
Supplement: SC-016-D4SC06893A-s001 [file SC-016-D4SC06893A-s001.pdf]

Supplementary Information (SI) for Chemical Science.
This journal is © The Royal Society of Chemistry 2024

Synthetic Anion Channels: Achieving Precise Mimicry of the Ion Permeation Pathway of CFTR in an Artificial System

Linlin Mao,†[a] Shuaimin Hou,†[a] Linlin Shi,[a] Jingjing Guo,*[b] Bo Zhu,[a] Yonghui Sun,*[a] Junbiao Chang,[a] and Pengyang Xin*[a]

[a] L. Mao, S. Hou, L. Shi, Dr. J. Guo, Dr. B. Zhu, Dr. Y. Sun, Prof. J. Chang, Dr. P. Xin.
State Key Laboratory of Antiviral Drugs, Pingyuan Laboratory, NMPA Key Laboratory for Research and Evaluation of Innovative Drug, School of Chemistry and Chemical Engineering
Henan Normal University
Xinxiang, 453007 (China)
E-mail: pyxin27@163.com, syonghui1994@163.com
[b] Prof. J. Guo
Centre in Artificial Intelligence Driven Drug Discovery, Faculty of Applied Sciences
Macao Polytechnic University
Macao, 999078 (China)
E-mail: jguo@mpu.edu.mo
† These authors contributed equally to this work

Contents

1. General: 3

2. Synthetic procedures and characterization data for **1-2**: 3

3. Procedures for planar lipid bilayer conductance experiments: 10

4. Ionophoric experiment with HPTS assay: 12

5. Cation transport experiment with HPTS assay: 12

6. Ion transport mechanism studies with HPTS assay: 13

7. Chloride transport activity with SPQ assay: 15

8. Anion transporting activity studies with HPTS assay: 16

9. Molecular dynamics simulations: 17

10. Reference 18

1. General:

All commercial reagents were used as supplied unless otherwise stated. Egg yolk L-α-phosphatidylcholine was obtained from Sigma-Aldrich as ethanol solution (100 mg/mL). 1,2-diphytanoyl-*sn*-glycero-3-phosphocholine (diPhyPC) was obtained from Avanti Polar Lipids as chloroform solution (10 mg/mL). The Fmoc-protected amino acids were obtained from GL Biochem (Shanghai) Ltd. The peptides were synthesized by PurePep Chorus automated peptide synthesizer. Mass spectra were recorded on Bruker MicroTOF II spectrometer. The ^{1}H and ^{13}C NMR spectra were recorded on commercial instruments (Bruker AVANCE III HD 600 MHz, Bruker AVANCE NEO 400 MHz) at 298 K. Chemical shifts were referenced to solvent residue. The conductance measurement on planar lipid bilayer was performed on Warner BC-535D Planar Lipid Bilayer Workstation. The fluorescent experiments on vesicles were performed on Varian Cary Eclipse fluorescence spectrophotometer.

2. Synthetic procedures and characterization data for 1-2:

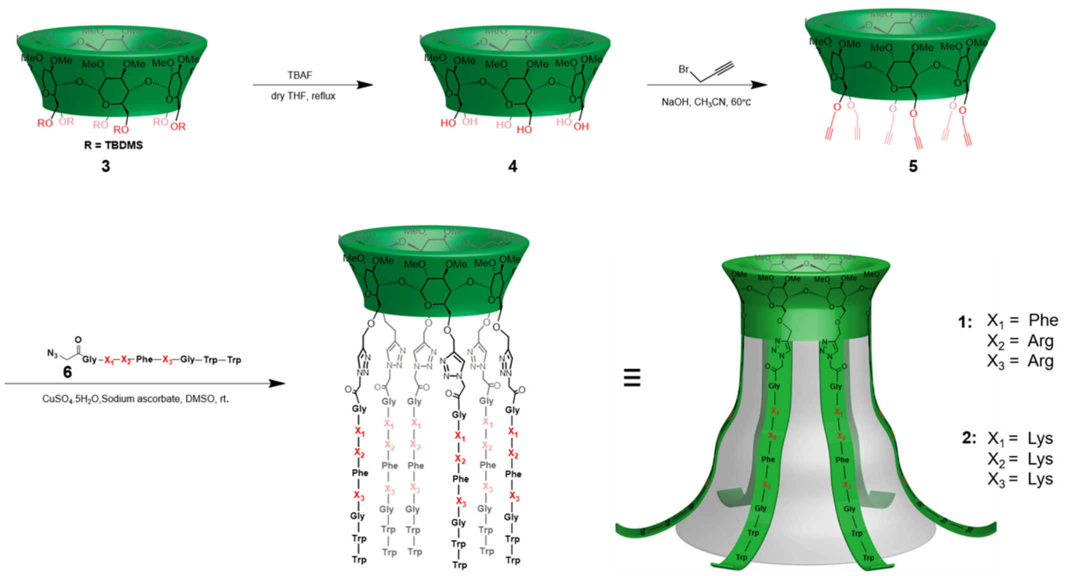

Compound 5. Compound **5** was synthesized according to the reported procedure.[1]

5: Yield: 95%. ^{1}H NMR ($CDCl_3$, 400 MHz) δ: 5.05 (d, J = 4.0, 6H), 4.35-4.19 (m, 12H), 4.04 (d, J = 8.0, 6H), 3.86-3.82 (m, 12H), 3.63 (s, 18H), 3.60-3.49 (m, 12H), 3.48 (s, 18H), 3.19-3.16 (m, 6H), 2.48 (t, J = 4.0, 6H). ^{13}C NMR ($CDCl_3$, 101 MHz) δ: 100.3, 82.6, 82.0, 81.2, 79.9, 77.3, 77.0, 76.7, 75.0, 71.0, 68.8, 61.9, 58.6, 57.8. HRMS: calcd for $C_{66}H_{96}NaO_{30}$ $[M+Na]^+$ 1391.5884, found 1391.5665.

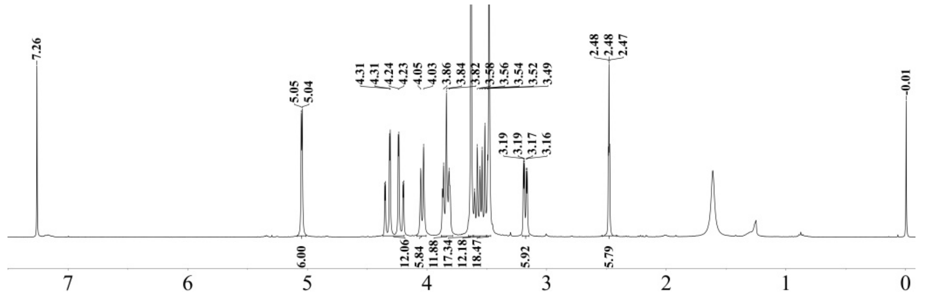

Fig. S1. ^{1}H NMR spectrum of **6** in $CDCl_3$.

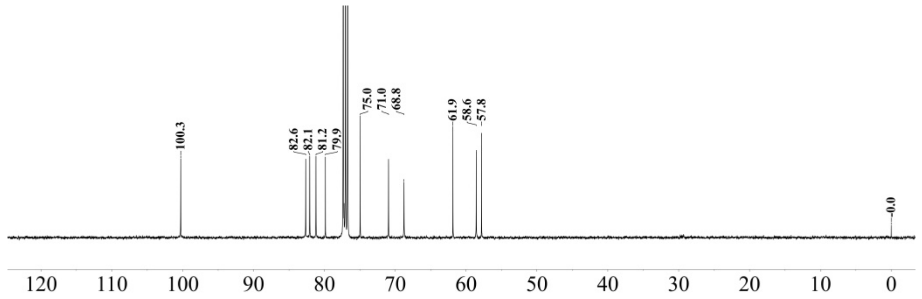

Fig. S2. ^{13}C NMR spectrum of **6** in $CDCl_3$.

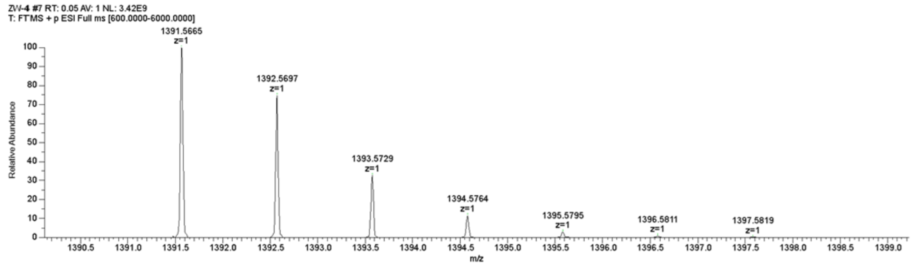

Fig. S3. HR-MS of **6**.

Compound 6a-6b. The peptides **6a-6b** were synthesized by standard Fmoc SPPS methods on a CEM PurePep Chorus automated peptide synthesizer. 2-chlorotrityl chloride resin Fmoc-protected amino acids were used for the solid phase peptide synthesis. Fmoc-protected amino acids, 2-chlorotrityl chloride resin, as well as coupling reagents were purchased from GL Biochem and used without further purification. Other reagents and solvents were purchased from Energy Chemical.

The PurePep Chorus peptide synthesizer was programmed to couple amino acids sequentially from the C to N terminus. 2-chlorotrityl chloride resin preloaded with Fmoc-protected amino acids (loading: 0.6 mmol/g) were used for the SPPS. Activation was achieved using HATU. All steps were executed at atmosphere. Detailed protocol used to program the automated peptide synthesizer is tabulated in Table S1.

Table S1. One cycle of the SPPS as performed using automated peptide synthesizer.

Entry	Operation	Solvent/Reagent	Temperature	Time (Repetitions)
1	Swelling	DMF	30°C	0.5 min (6x)
2	Fmoc removal	20% Piperidine / DMF	30°C	6 min (2x)
3	Wash	DMF	30°C	0.5 min (6x)
4	Activation	NMM (*N*-methyl morphorphine) in DMF (0.4 M)	30°C	6 min (1x)
5	Coupling	Amino acid (3eq.) : HBTU : NMM = 1:1:2	50°C	60 min (1x)
6	Wash	DMF	30°C	0.5 min (6x)

Upon completion of the peptide synthesis process, the resin was relocated into a designated vessel for further peptide synthesis operations. The HATU (1 eq.), N_3-CH_2COOH (2 eq.), NMM (0.4 M in DMF, 5 mL) were added to a flask and stirred for 5 min. Above mixture was introduced into the peptide synthesis vessel and then agitated for an additional 8 hours at ambient temperature. After completion of the reaction, the resin was collected by filtration and washed with DMF (6 × 5mL), methanol (6 × 5mL), dichloromethane (6 × 5 mL) successively. Then, the cleavage reagent (TFA : TIS : H_2O = 95 : 2.5 : 2.5) was added to the synthesis vessel and reacted for 6 hours at room temperature. The mixture was filtered and the filtrate was collected. Following cleavage the cleavage reagent was reduced in volume to ≈ 5 mL using a flow of nitrogen. Addition of diethyl ether (≈ 45 mL) resulted in a precipitate that was recovered via centrifugation and redissolved in a 1:1 solution of acetonitrile in water before freeze-drying, to yield the crude peptide as white solid.

The synthesized peptide was purified via preparative reverse-phase HPLC on an Agilent 1260 system, utilizing an SB-C18 column (21.2 × 250 mm, 7 μm). The purification process employed two eluents: A (100% water with 0.1% TFA) and B (100% acetonitrile with 0.1% TFA). The purification progress was monitored by UV detection at 215 nm. The linear gradient from 95% A and 5% B to 5% A and 95% B over 40 min was used in the purification processes. All analytic HPLC (Agilent 1260) were run with a SB-C18 column (4.6 × 150 mm, 5 μm) using the identical linear gradient with above purification processes.

6a: N_3-CH_2-CO-Gly-Phe-Arg-Phe-Arg-Gly-Trp-Trp-COOH

HPLC retention time: 9.92 min, purity: 87 %. HRMS: calcd for $C_{58}H_{73}N_{19}O_{10}$ $[M+2H]^{2+}$ 597.7888, found 597.7940.

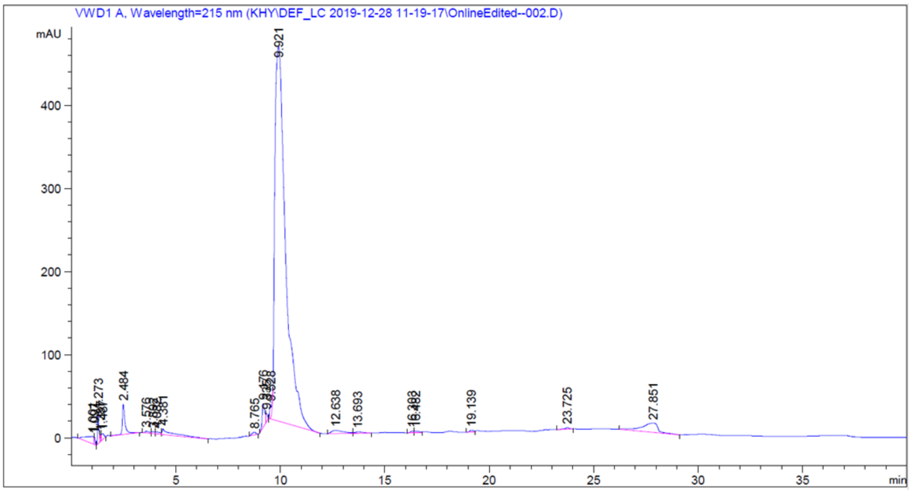

Fig. S4. HPLC analytic trace of **6a**.

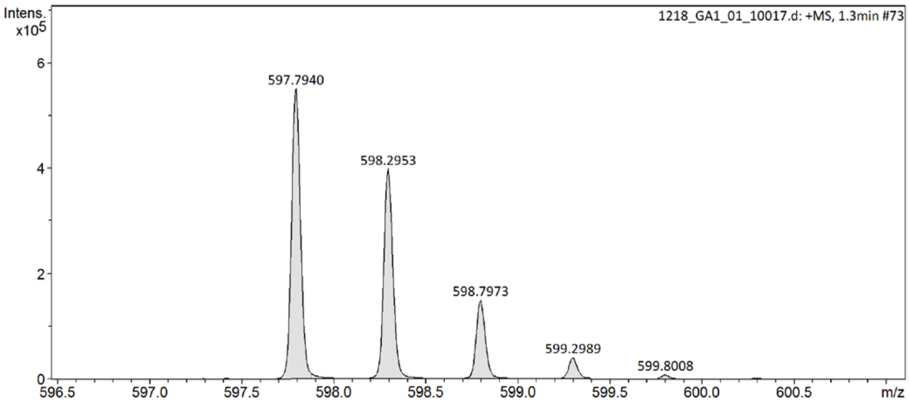

Fig. S5. HR-MS of **6a**.

6b: N_3-CH_2-CO-Gly-Lys-Lys-Phe-Lys-Gly-Trp-Trp-COOH

HPLC retention time: 7.56 min, purity: 95 %. HRMS: calcd for $C_{55}H_{74}N_{16}NaO_{10}$ $[M+Na]^+$ 1141.5666, found 1141.5146.

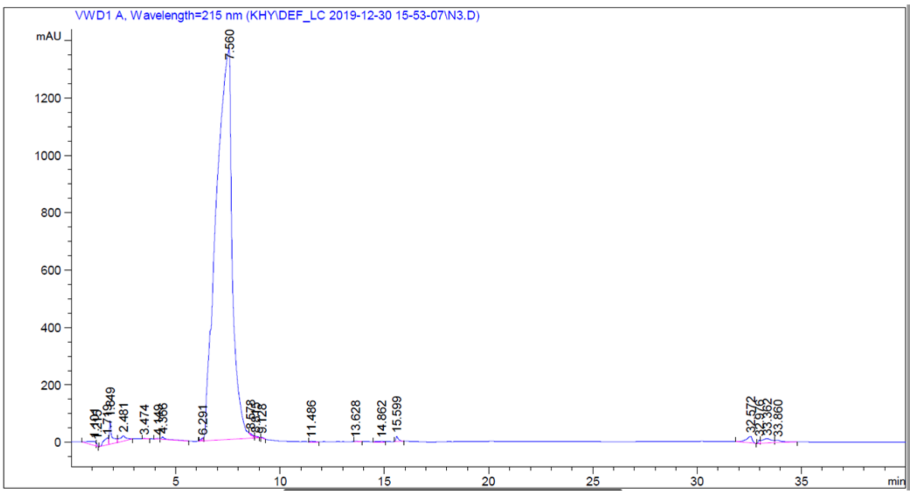

Fig. S6. HPLC analytic trace of **6b**

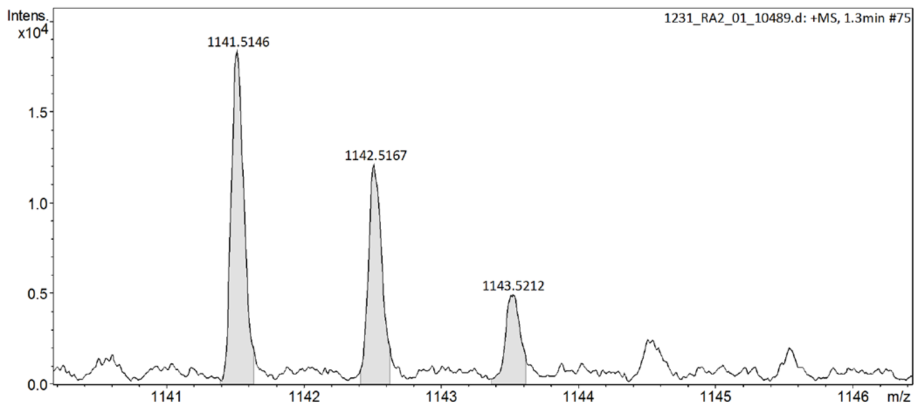

Fig. S7. HR-MS of **6b**.

Compound 1. Compound **5** (30.0 mg, 0.02 mmol) and Compound **6a** (250.0 mg, 0.22 mmol) was dissolved in DMSO (12 mL). Sodium ascorbate (89.1 mg, 0.45 mmol) and $CuSO_4•5H_2O$ (44.9 mg, 0.18 mmol) was added, and the reaction mixture was stirred at 100 °C for 48 h. 10 portions of water was added to the reaction mixture, and the obtained mixture was freeze-dried. The crude product was purified via preparative reverse-phase HPLC on an Agilent 1260 system, utilizing an SB-C18 column (21.2 × 250 mm, 7 μm). The purification process employed two eluents: A (100% water with 0.1% TFA) and B (100% acetonitrile with 0.1% TFA). The purification progress was monitored by UV detection at 215 nm. The linear gradient from 95% A and 5% B to 5% A and 95% B over 30 min was used in the purification processes. All analytic HPLC (Agilent 1260) were run with a SB-C18 column (4.6 × 150 mm, 5 μm) using the identical linear gradient with above purification processes.

1: Yield: 32%. HPLC retention time: 18.11 min, purity: 93 %. ^{1}H NMR (DMSO-*d6*, 600 MHz) δ: 10.80 (d, *J* = 18.0, 12H), 8.34-8.31 (m, 9H), 8.21-8.11 (m, 18H), 8.04 (s, 12H), 7.95 (s, 6H), 7.92-7.86 (m, 12H), 7.78 (br, 12H), 7.62-7.59 (m, 12H), 7.50 (d, *J* = 6.0, 6H), 7.45-7.41 (m, 24H), 7.33-7.28 (m, 18H), 7.23-7.18 (m, 30H), 7.14 (s, 6H), 7.13-7.12 (m, 12H), 7.06-7.04 (m, 12H), 6.99-6.96 (m, 12H), 4.64-4.51 (m, 30H), 4.27-4.22 (m, 18H), 3.84-3.72 (m, 24H), 3.51-3.50 (m, 18H), 3.18-3.12 (m, 24H), 3.12 (br, 30H), 2.94-2.89 (m, 24H), 2.84-2.77 (m, 12H), 2.73 (s, 12H), 2.54 (s, 24H), 2.41 (d, *J* = 6.0, 6H), 2.04-1.95 (m, 12H), 1.63 (br, 12H), 1.47 (br, 12H), 1.40-1.39 (m, 12H). ^{13}C NMR (DMSO-*d6*, 150 MHz) δ: 175.6, 172.7, 171.4, 136.0, 129.2, 128.0, 127.3, 126.2, 124.3, 123.7, 120.9, 120.8, 118.4, 118.2, 111.4, 111.3, 110.0, 86.3, 73.0, 72.2, 69.8, 65.8, 43.1, 40.4, 38.2, 33.3, 31.3, 29.8, 29.0, 28.7, 28.6, 28.3, 24.0, 23.3, 22.4, 22.1, 17.6, 14.0, 13.9, 12.3, 10.8. HRMS: calcd for $C_{414}H_{522}N_{114}K_2O_{90}$ $[M+2K+NH_4]^{3+}$ 2876.9970, found 2876.7928.

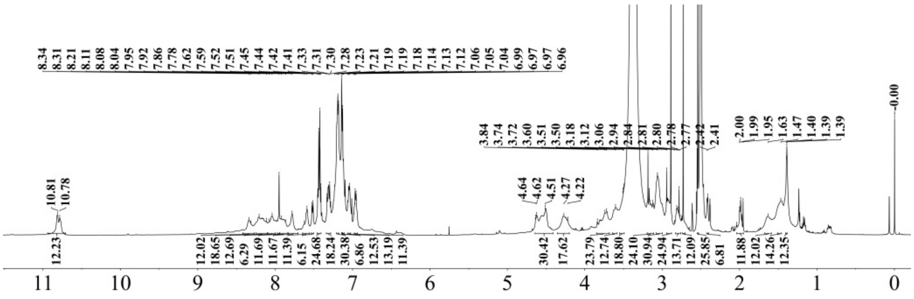

Fig. S8. ^{1}H NMR spectrum of **1** in DMSO-*d6*.

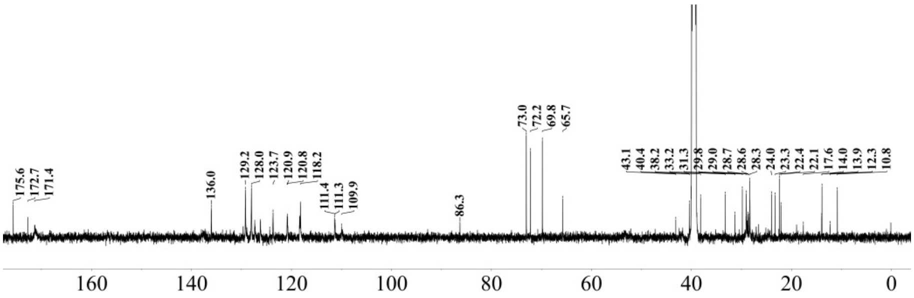

Fig. S9. ^{13}C NMR spectrum of **1** in DMSO-*d6*.

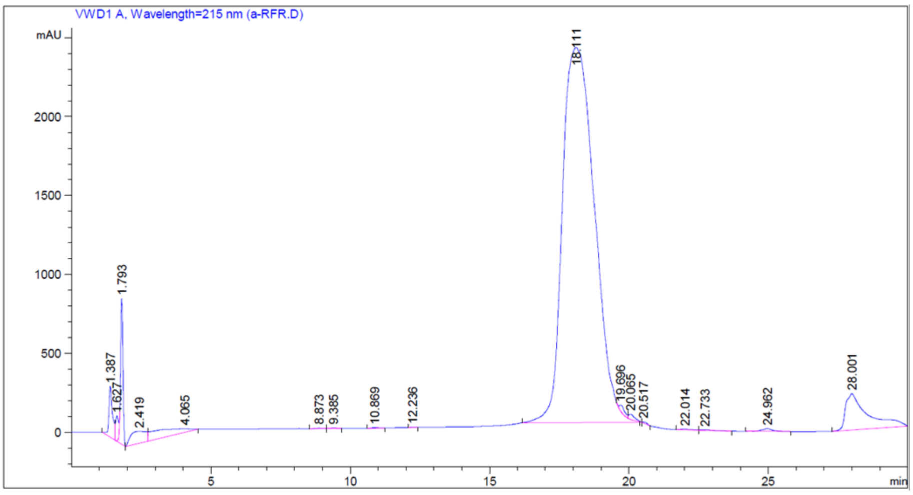

Fig. S10. HPLC analytic trace of **1**.

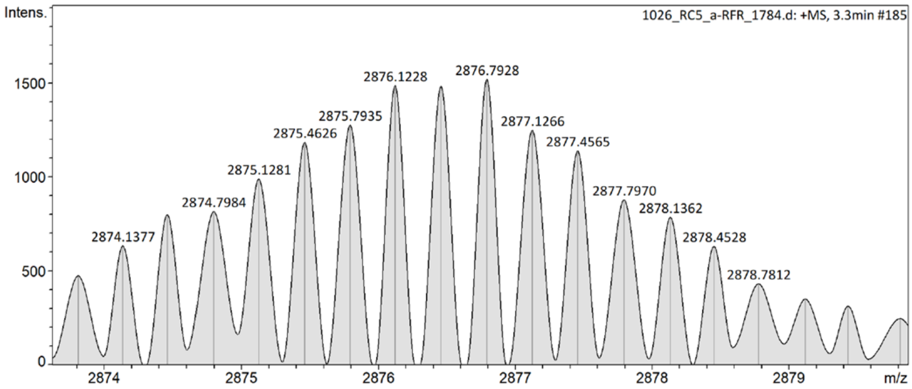

Fig. S11. HR-MS of **1**.

Compound 2. Compound **2** was synthesized from compound **5** and **6b** according to the same procedure with compound **1**.

2: Yield: 43%. HPLC retention time: 17.94 min, purity: 92 %. ^{1}H NMR (DMSO-*d6*, 400 MHz) δ: 12.62 (s, 6H), 10.82 (d, *J* = 12.0, 12H), 8.55 (s, 6H), 8.34 (s, 12H), 8.12 (br, 12H), 8.02-8.94 (m, 36H), 7.72 (br, 24H), 7.59 (d, *J* = 8.0, 6H), 7.52 (d, *J* = 8.0, 6H), 7.32 (t, *J* = 8.0, 12H), 7.26 (s, 6H), 7.19 (br, 24H), 7.15-7.14 (m, 12H), 7.12 (s, 6H), 7.07-7.03 (m, 12H), 7.01 (s, 6H), 6.99-6.94 (m, 12H), 5.13 (s, 6H), 4.98 (s, 6H), 4.61 (s, 6H), 4.51 (s, 12H), 4.22 (br, 18H), 3.88 (s, 6H), 3.78 (br, 12H), 3.69 (s, 6H), 3.63 (s, 6H), 3.21 (d, *J* = 4.0, 6H), 3.17 (s, 6H), 3.12 (s, 6H), 3.09 (s, 6H), 3.07-3.01 (m, 18H), 2.97-2.87 (m, 12H), 2.73 (br, 18H), 1.62-1.27 (m, 180H). ^{13}C NMR (DMSO-*d6*, 101 MHz) δ: 173.6, 172.3, 171.9, 171.4, 170.2, 168.8, 166.3, 161.6, 158.8, 144.4, 138.0, 136.7, 136.5, 136.5, 129.6, 128.5, 127.9, 127.7, 126.7, 125.9, 125.4, 124.2, 121.7, 121.4, 121.3, 118.9, 118.7, 112.1, 111.9, 111.8, 110.3, 110.1, 106.7, 99.4, 81.8, 63.5, 61.5, 57.6, 55.7, 54.0, 53.5, 53.2, 52.9, 51.9, 42.4, 37.4, 32.4, 32.1, 31.7, 31.3, 30.2, 29.8. 29.1, 28.4, 27.5, 27.1, 22.9, 22.6. HRMS: calcd for $C_{396}H_{542}N_{96}NaO_{90}$ $[M+Na+2H]^{3+}$ 2703.0267, found 2704.7214.

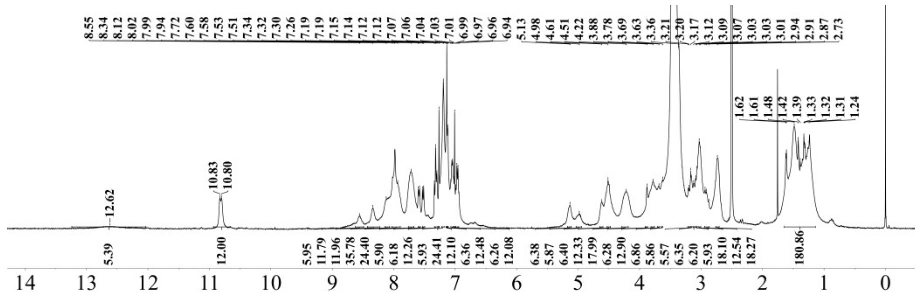

Fig. S12. ^{1}H NMR spectrum of **2** in DMSO-*d6*.

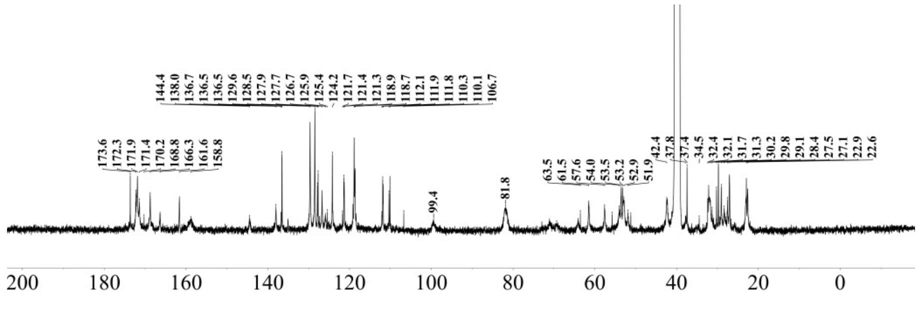

Fig. S13. ^{13}C NMR spectrum of **2** in DMSO-*d6*.

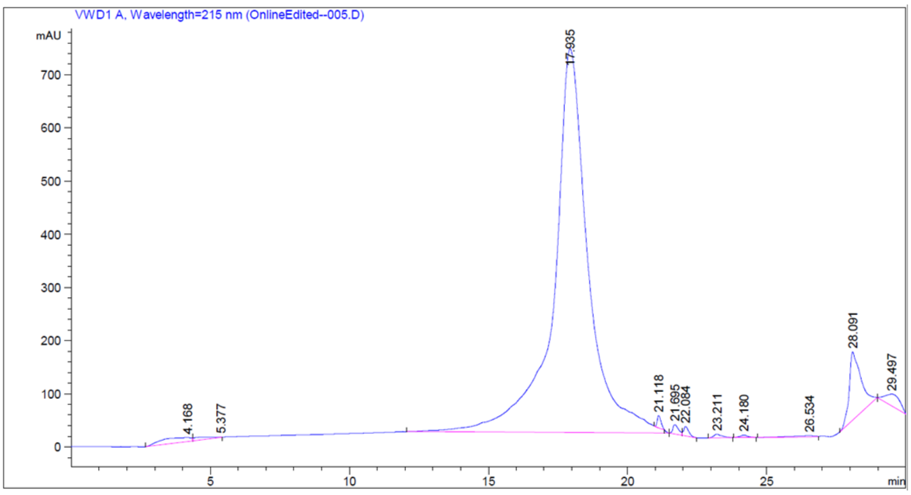

Fig. S14. HPLC analytic trace of **2.**

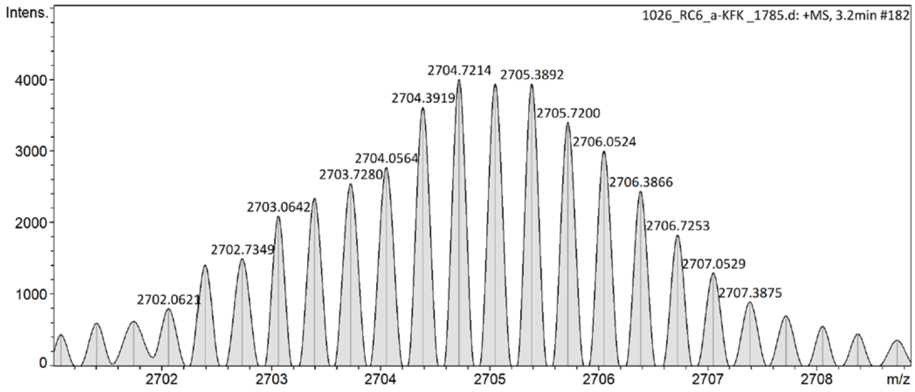

Fig. S15. HR-MS of **2**.

3. Procedures for planar lipid bilayer conductance experiments:

The solution of diPhyPC in chloroform (10 mg/mL, 20 μL) was evaporated with nitrogen gas to form a thin film and re-dissolved in *n*-decane (8 μL). The lipid solution (0.5 μL) was injected on to the aperture (diameter = 200 μm) of the Delrin® cup (Warner Instruments, Hamden, CT) and then evaporated with nitrogen gas. In a typical experiment for measurement of the channel conductance for an ion, the chamber (*cis* side) and the Delrin cup (*trans* side) were filled with aqueous KCl solution (1.0 M, pH = 7.0). Ag-AgCl electrodes were applied directly to the two solutions and the *cis* one was grounded. Planar lipid bilayer was formed by painting the lipids solution (1.0 μL) around the pretreated aperture and by judgment of capacitance (80-120 pF). Membrane currents were measured using a Warner BC-535D bilayer clamp amplifier and were collected by PatchMaster (HEKA) with sample interval at 10 kHz and then filtered with an 8-pole Bessel filter at 1 kHz (HEKA). The data were analyzed by FitMaster (HEKA) with a digital filter at 110 Hz. All data were analyzed by the software pClamp 10.6.

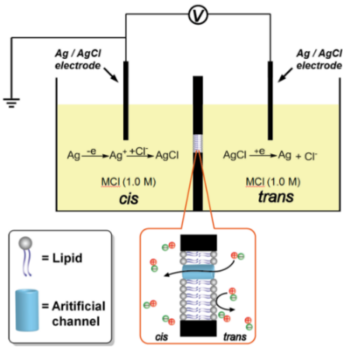

Fig. S16. Schematic representation for the patch clamp experiments with planar lipid bilayer. The redox reactions on both Ag/AgCl electrodes are inserted to illustrate the nature of charge balance during ion transmembrane transport.

For the single-channel conductance measurement, two chambers were charged with KCl (1 M, 1 mL). And the solution of compound **1-2** in DMSO (0.1 mM, 2 μL) was added to the *cis* compartment and the solution was stirred for 5 min.

For the measurement of the transport selectivity of K^+ over Cl^-, the KCl solutions (0.5 M and 1.0 M) were added to the both side of the bilayer (diPhyPC), *cis* chamber: KCl (1.0 M), *trans* chamber: KCl (0.5 M). The solution of compound **1-2** in DMSO (0.1 mM, 2 μL) was added to the *cis* compartment and the solution was stirred for 5 min. The measured reversal potentials obtained from the *I-V* plots needed adjustment, accounting for the redox potential produced by disparate voltage drops at the electrode-solution interface in different electrolyte concentrations.[1] The redox potential (E_{redox}) was calculated from the equation:

$$E_{redox} = \frac{RT}{zF} \ln \frac{\gamma_{C_H} c_H}{\gamma_{C_L} c_L} \qquad (1)$$

where the R, T, z, F, and γ are the gas constant, temperature, charge number, Faraday constant, and mean activity coefficient. When *cis/trans* = 1.0 M/0.5 M KCl, γ_{C_H} = 0.604, γ_{C_L} = 0.649, c_H = 1.0, c_L = 0.5.

Then, the corrected reversal voltage (V_r) was calculated from the equation:

$$V_r = V_{measured} - E_{redox} \qquad (2)$$

where the $V_{measured}$ is the measured reversal voltage.

The P_{K^+}/P_{Cl^-} values were calculated from the equation derived from Goldman-Hodgin-Katz equation:

(3)

$$P_{K^+}/P_{Cl^-} = \left[a_{K,cis} - a_{K,trans}\exp\left(\frac{-V_r F}{RT}\right)\right] / \left[a_{Cl,cis}\exp\left(\frac{-V_r F}{RT}\right) - a_{Cl,trans}\right]$$

where $a_{K,\ cis}$ and $a_{K,\ trans}$ are the activities of K^+ in the *cis* and the *trans* chambers, $a_{Cl,\ cis}$ and $a_{Cl,\ trans}$ the same for Cl^-.

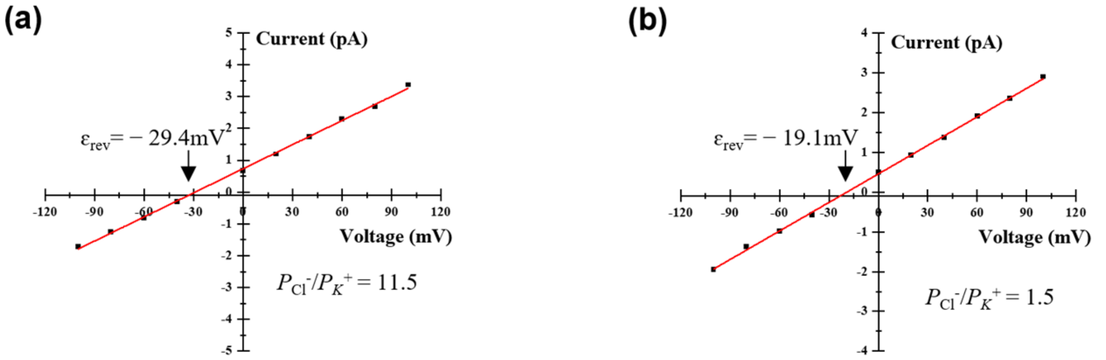

Fig. S17. *I–V* plots of **1-2** by using unsymmetrical solution at both side of the bilayer. *cis* chamber: KCl (1.0 M), *trans* chamber: KCl (0.5 M). (a) **1**, (b) **2**.

4. Ionophoric experiment with HPTS assay:

(a) Preparation of HPTS containing large unilamellar vesicles (LUVs): Egg yolk L-α-phosphatidylcholine (EYPC, 20 mg) a was diluted with $CHCl_3$ (5.0 mL), the solution was evaporated under reduced pressure on a rotary evaporator (40 °C) to give a thin film, and the resulting thin film was dried under high vacuum for 3 h. The lipid film was hydrated with HEPES buffer solution (2 mL, HEPES (10 mM), NaCl (100 mM), pH = 7.0) containing 8-hydrox-ypyrene-1,3,6-trisulfonic acid (HPTS, 0.1 mM) at 40 °C for 2 h to give a milky suspension. The resulting suspension was subjected to ten freeze-thaw cycles by using liquid N_2 to freeze and warm water bath to thaw. The suspension was extruded via polycarbonate membrane (0.2 μm) suspension nine times and then dialyzed with membrane tube (MWCO = 14000) against the same HEPES buffer solution (200 mL, without HPTS) for six times to remove un-entrapped HPTS and produce vesicle suspension ([lipid] = 13.3 mM).

(b) Fluorescent experiments: The prepared vesicle suspension (13.3 mM, 30 μL) was added to a HEPES buffer solution (1.97 mL, HEPES (10 mM), Na_2SO_4(67 mM), pH = 8.0) to create a pH gradient for ion transport study. To the cuvette, the solution of compound **1-2** in DMF (5 μL) was added to reach a required channel concentration (molar ratio relative to lipid, represented by x) with gentle stirring. Fluorescent intensity (I_t) was continuously monitored at 510 nm (excitation at 460 nm) in 300 seconds. Then, Triton aqueous solution (20%, 10 μL) was added with gentle stirring. The intensity was monitored until the fluorescent intensity (I_∞) did not change. The collected data were then normalized into the fractional change in fluorescence given by $(I_t-I_0)/(I_\infty-I_0)$, where I_0 is the initial intensity.

5. Cation transport experiment with HPTS assay:

(a) Preparation of HPTS containing large unilamellar vesicles (LUVs): The vesicles (2 mL, HEPES (10 mM), Na_2SO_4 (100 mM), pH = 7.0) were prepared by the following protocol as stated above.

(b) Cation transport activity by HPTS assay: The prepared vesicle suspension (13.3 mM, 30 μL) was added to a HEPES buffer solution (1.97 mL, HEPES (10 mM), Na_2SO_4 (100 mM), pH = 8.0) to create a pH gradient for ion transport study. A solution of compound in DMF was then injected into the suspension under gentle stirring to reach a final compound concentration of compound **1-2** (x = 0.63%, molar ratio relative to lipid, represented by x), carbonyl cyanide-4 (trifluoromethoxy)phenylhydrazone (FCCP) (1×10^{-6} M), and gramicidin A (gA) (2×10^{-6} M). Fluorescent intensity (I_t) was continuously monitored at 510 nm (excitation at 460 nm) in 300 seconds. Then, Triton aqueous solution (20%, 10 μL) was added with gentle stirring. The intensity was monitored until the fluorescent intensity (I_∞) did not change. The collected data were then normalized into the fractional change in fluorescence given by $(I_t\text{-}I_0)/(I_\infty\text{-}I_0)$, where I_0 is the initial intensity.

(c) Cation selectivity using the HPTS Assay: The prepared vesicle suspension (13.3 mM, 30 μL) was added to a HEPES buffer solution (1.97 mL, HEPES (10 mM), M_2SO_4 ((100 mM), M = Cs^+, Rb^+, K^+, Na^+, Li^+, pH = 8.0) to create a pH gradient for ion transport study. A solution of compound **1-2** (x = 0.63%) in DMF was then injected into the suspension under gentle stirring. Fluorescent intensity (I_t) was continuously monitored at 510 nm (excitation at 460 nm) in 300 seconds. Then, Triton aqueous solution (20%, 10 μL) was added with gentle stirring. The intensity was monitored until the fluorescent intensity (I_∞) did not change. The collected data were then normalized into the fractional change in fluorescence given by $(I_t\text{-}I_0)/(I_\infty\text{-}I_0)$, where I_0 is the initial intensity.

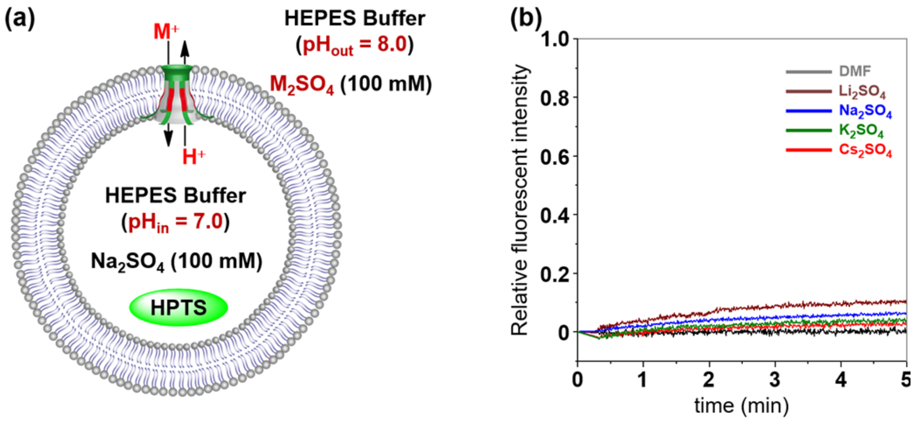

Fig. S18. (a) Schematic representation for the HPTS assay. (b) Changes in the relative fluorescence intensity of HPTS (λ_{ex} = 460 nm, λ_{em} = 510 nm) in vesicles with time in the presence of **2**.

6. Ion transport mechanism studies with HPTS assay:

(a) Preparation of HPTS containing large unilamellar vesicles (LUVs): The vesicles (2 mL, HEPES (10 mM), NaCl (100 mM), pH = 7.0) were prepared by the following protocol as stated above.

(b) Ion transport activity in the presence of FCCP: The fluorescence intensity of compound **1-2** was measured in the absence and presence of FCCP. The prepared vesicle suspension (13.3 mM, 30 μL) was added to a HEPES buffer solution (1.97 mL, HEPES (10 mM), Na_2SO_4 (67 mM), pH = 8.0) to create a pH gradient for ion transport study. The solution of compound **1** or **2** (x = 0.63%, molar ratio relative to lipid, represented by x), carbonyl cyanide 4-(trifluoromethoxy) phenyl hydrazone (FCCP, 5×10^{-7} M), and a mixture of compound **1-2**/FCCP were added to the cuvette separately. Fluorescent intensity (I_t) was continuously monitored at 510 nm (excitation at 460 nm) in 300 seconds. Then, Triton aqueous solution (20%, 10 μL) was added with gentle stirring. The intensity was monitored until the fluorescent intensity (I_∞) did not change. The collected data were then normalized into the fractional change in fluorescence given by $(I_t-I_0)/(I_\infty-I_0)$, where I_0 is the initial intensity.

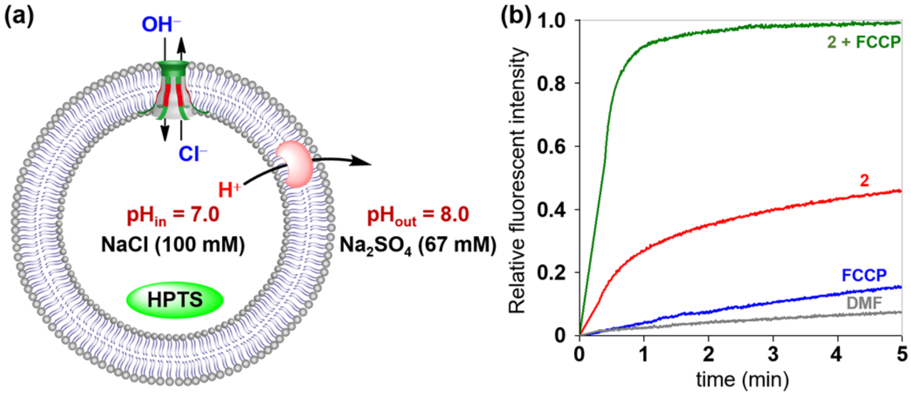

Fig. S19. (a) Schematic representation for the HPTS assay. (b) Changes in the relative fluorescence intensity of HPTS (λ_{ex} = 460 nm, λ_{em} = 510 nm) in vesicles with time in the presence of **2** or FCCP.

(c) Ion transport activity in the presence of VA (valinomycin): The fluorescence intensity of compound **1-2** was measured in the absence and presence of VA. The prepared vesicle suspension (13.3 mM, 30 μL) was added to a HEPES buffer solution (1.97 mL, HEPES (10 mM), K_2SO_4 (67 mM), pH = 8.0) to create a pH gradient for ion transport study. The solution of compound **1** or **2** (x = 0.63%, molar ratio relative to lipid, represented by x), valinomycin (VA, 5×10^{-10} M), and a mixture of compound **1-2**/VA were added to the cuvette separately. Fluorescent intensity (I_t) was continuously monitored at 510 nm (excitation at 460 nm) in 300 seconds. Then, Triton aqueous solution (20%, 10 μL) was added with gentle stirring. The intensity was monitored until the fluorescent intensity (I_∞) did not change. The collected data were then normalized

into the fractional change in fluorescence given by (I_t-I_0)/(I_∞-I_0), where I_0 is the initial intensity.

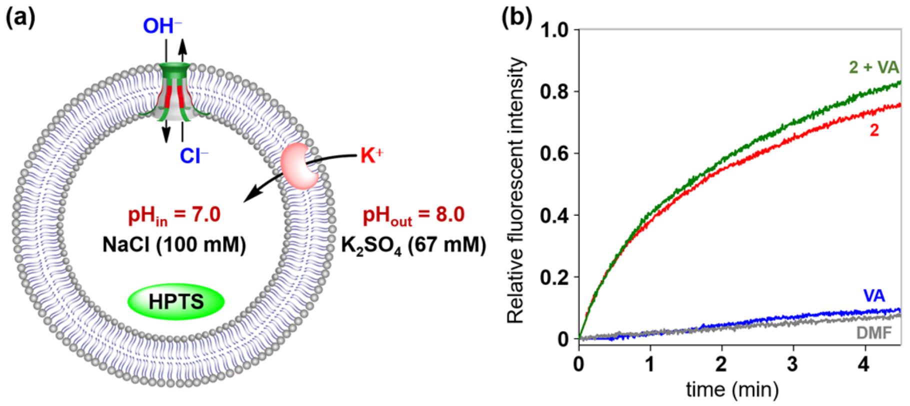

Fig. S20. (a) Schematic representation for the HPTS assay. (b) Changes in the relative fluorescence intensity of HPTS (λ_{ex} = 460 nm, λ_{em} = 510 nm) in vesicles with time in the presence of **2** or VA.

7. Chloride transport activity with SPQ assay:

(a) Preparation of SPQ (6-methoxy-N-(3-sulfopropyl)quinolinium) containing large unilamellar vesicles (LUVs): Egg yolk L-α-phosphatidylcholine (EYPC, 20 mg) a was diluted with $CHCl_3$ (5.0 mL), the solution was evaporated under reduced pressure on a rotary evaporator (40 °C) to give a thin film, and the resulting thin film was dried under high vacuum for 3 h. The lipid film was hydrated with $NaNO_3$ solution (1 mL, 225 mM) containing a Cl^--sensitive dye 6-methoxy-N-(3-sulfopropyl) quinolinium (SPQ, 0.5 mM) at 40 °C for 2 h to give a milky suspension. The resulting suspension was subjected to twelve freeze-thaw cycles by using liquid N_2 to freeze and warm water bath to thaw. The suspension was extruded via polycarbonate membrane (0.2 μm) suspension nine times and then dialyzed with membrane tube (MWCO = 14000) against the same HEPES buffer solution (200 mL, without SPQ) for five times to remove un-entrapped SPQ and produce vesicle suspension ([lipid] = 13.3 mM).

(b) Ion transport activity by SPQ assay: The prepared vesicle suspension (13.3 mM, 30 μL) was added to a NaCl solution (1.97 mL, 225 mM) to create extravesicular chloride gradient. To the cuvette, the solution of compound **1-2** in DMF (5 μL) was added to reach a required channel concentration with gentle stirring. Fluorescent intensity (I_t) was continuously monitored at 443 nm (excitation at 344 nm) in 600 seconds. Then, Triton aqueous solution (20%, 10 μL) was added with gentle stirring. The intensity was monitored until the fluorescent intensity (I_∞) did not change. The collected data were then normalized into the fractional change in fluorescence given by (I_t-I_0)/(I_∞-I_0), where I_0 is the initial intensity.

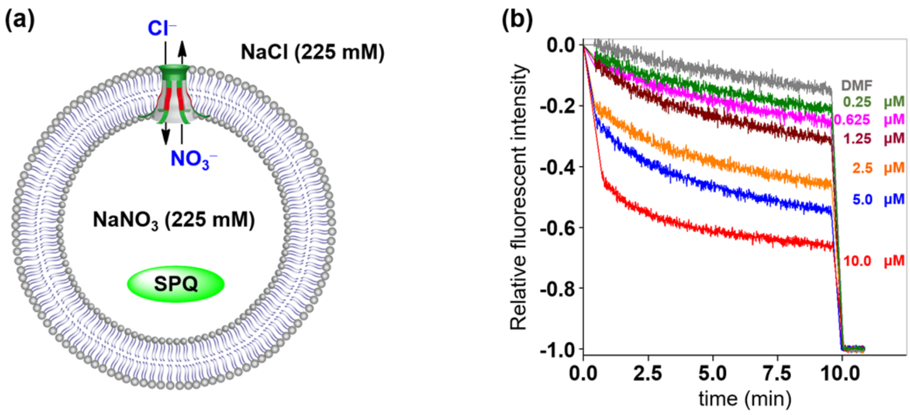

Fig. S21. (a) Schematic representation for the SPQ assay. (b) Changes in the relative fluorescence intensity of SPQ (λ_{ex} = 344 nm, λ_{em} = 443 nm) in vesicles with time in the presence of **2**.

8. Anion transporting activity studies with HPTS assay:

(a) Preparation of HPTS containing large unilamellar vesicles (LUVs): Egg yolk L-α-phosphatidylcholine (EYPC, 20 mg) a was diluted with $CHCl_3$ (5.0 mL), the solution was evaporated under reduced pressure on a rotary evaporator (40 °C) to give a thin film, and the resulting thin film was dried under high vacuum for 3 h. The lipid film was hydrated with HEPES buffer solution (2 mL, HEPES (10 mM), NaX (100 mM), X^- = Cl^-, Br^-, I^-, NO_3^-, SO_4^{2-}, pH = 7.0) containing HPTS (0.1 mM) at 40 °C for 2 h to give a milky suspension. The resulting suspension was subjected to ten freeze-thaw cycles by using liquid N_2 to freeze and warm water bath to thaw. The suspension was extruded via polycarbonate membrane (0.2 μm) suspension nine times and then dialyzed with membrane tube (MWCO = 14000) against the same HEPES buffer solution (200 mL, without HPTS) for six times to remove un-entrapped HPTS and produce vesicle suspension ([lipid] = 13.3 mM).

(b) Ion transport activity by HPTS assay: The prepared vesicle suspension (13.3 mM, 30 μL) was added to a HEPES buffer solution (1.97 mL, HEPES (10 mM), Na_2SO_4(67 mM), pH = 8.0) to create a pH gradient for ion transport study. To the cuvette, the solution of compound **1-2** in DMF (x = 0.63%, molar ratio relative to lipid, represented by x) was added with gentle stirring. Fluorescent intensity (I_t) was continuously monitored at 510 nm (excitation at 460 nm) in 300 seconds. Then, Triton aqueous solution (20%, 10 μL) was added with gentle stirring. The intensity was monitored until the fluorescent intensity (I_∞) did not change. The collected data were then normalized into the fractional change in fluorescence given by $(I_t-I_0)/(I_\infty-I_0)$, where I_0 is the initial intensity.

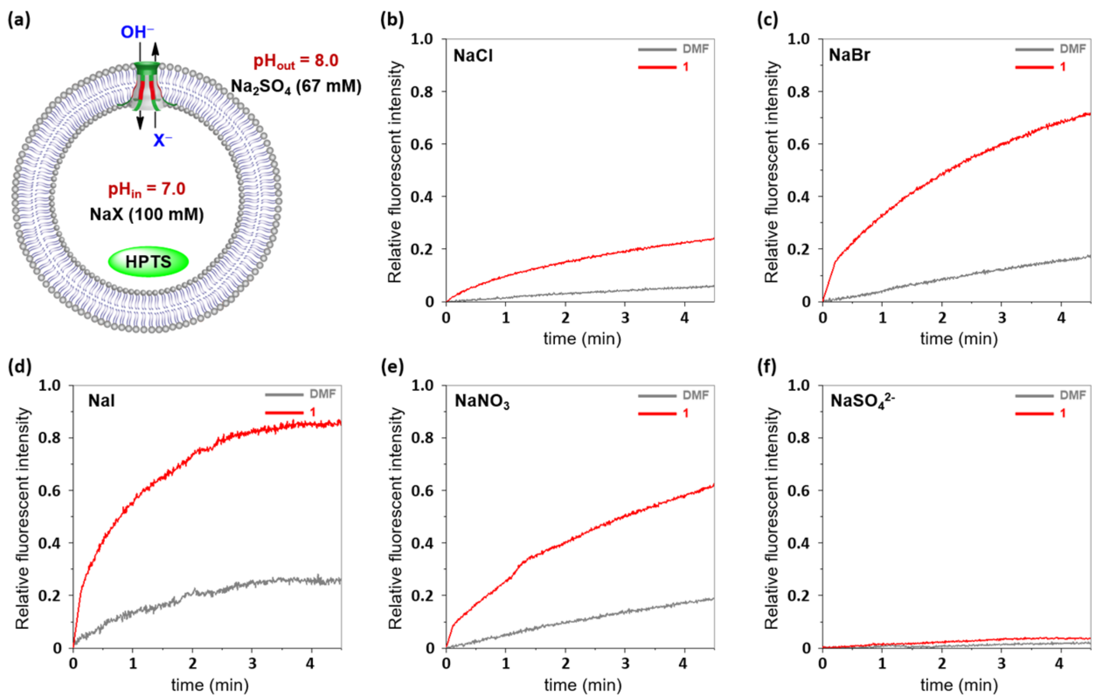

Fig. S22. (a) HPTS assay with varied internal anionic ions (X^{-} = Cl^{-}, Br^{-}, I^{-}, NO_3^{-}, SO_4^{2-}). (b)-(f) Changes in the relative fluorescence intensity of HPTS (λ_{ex} = 460 nm, λ_{em} = 510 nm) in vesicles with time in the presence of **1**.

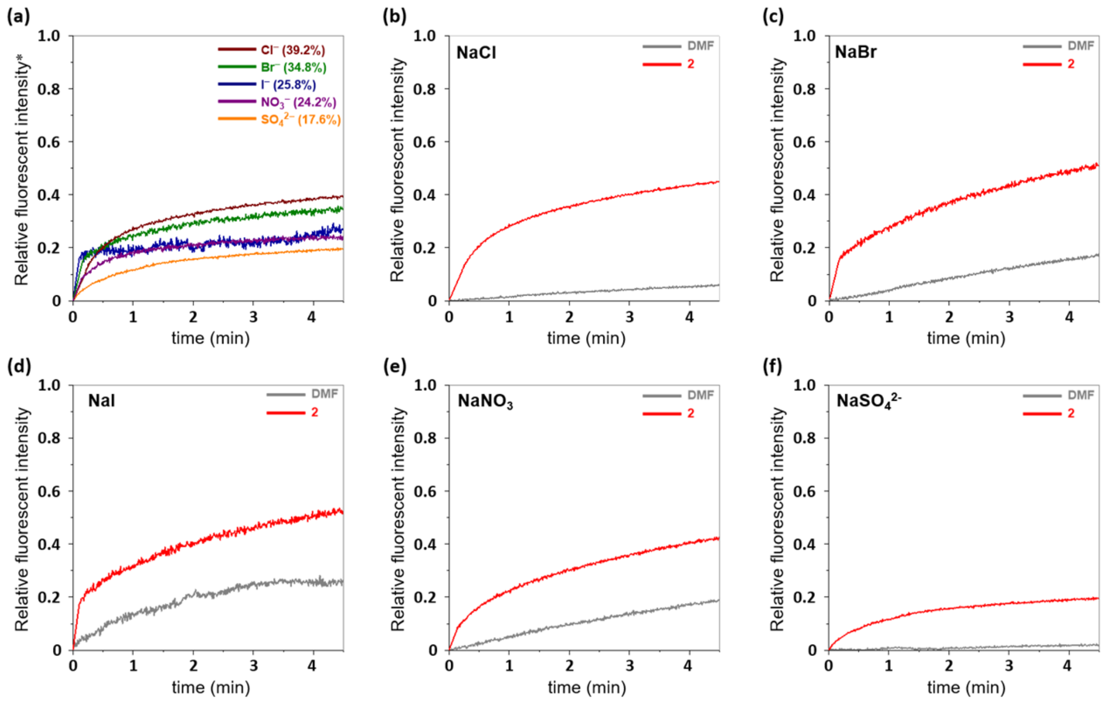

Fig. S23. (a) Changes in the relative fluorescence intensity of HPTS (λ_{ex} = 460 nm, λ_{em} = 510 nm) in vesicles with time in the presence of **2** (* baselinecorrected). (b)-(f) Changes in the relative fluorescence intensity of HPTS in vesicles with time in the presence of **2**.

9. Molecular dynamics simulations:

The initial configuration of the channel was constructed based on our previous

work,[2] and inserted into a bilayer with 256 diPhyPC lipids generated from the CHARMM-GUI online server,[3] and the overlapping lipids were removed. After solvation with TIP3P waters, the system was neutralized with 1.0 mol/L KCl, yielding a system with approximately 85,000 atoms. The cgenff.paramchem.org server was used to build the force field parameters of the α-CD derivative, and the CHARMM 36 force field was used for the peptides.[4-5]

Firstly, the channel was held fixed while lipids and water were allowed to equilibrate for a 150-ps NVT equilibration following a 10,000-step minimization, performed in 4 steps: 1-2) fix the protein backbone and heavy atoms of the rest, and apply a harmonic potential of 5 and 2 kcal/mol to lipid-head heavy atoms; 3) apply restraints to backbone atoms of the channel and lipid heads; 4) restrain important channel atoms with 1 kcal/mol force constant. The final model was obtained after a 10-ns NPT production run following the structural minimization performed in NAMD 3.0,[6] and then used as the starting point for the constant velocity steered molecular dynamics (SMD) simulation. In this step, the translocation coordinate (Z) was defined as the normal to the bilayer surface. During the SMD simulation, a K^+ ion was pulled through the channel at a constant velocity of 0.5 Å/ns. The force constant for the spring connecting the K^+ ion and the imaginary atom was set to 5.0 kcal/mol·Å^2. Additionally, the backbone atoms of α-CD were restrained in the *Z* direction (the SMD direction) with a force constant of 5.0 kcal/mol·A^2 to prevent the system from drifting.

To understand the permeation of K^+ ions through the channel, we determined the potentials of mean force (PMF) along the channel axis using adaptive biasing forces. The simulations were conducted in six windows, each spanning 5 Å along the Z direction, which adequately covered the entire length of the channel. Each window was subjected to a production run of 20 ns, culminating in a total simulation time of 120 ns. For Cl^-, the same procedures were used.

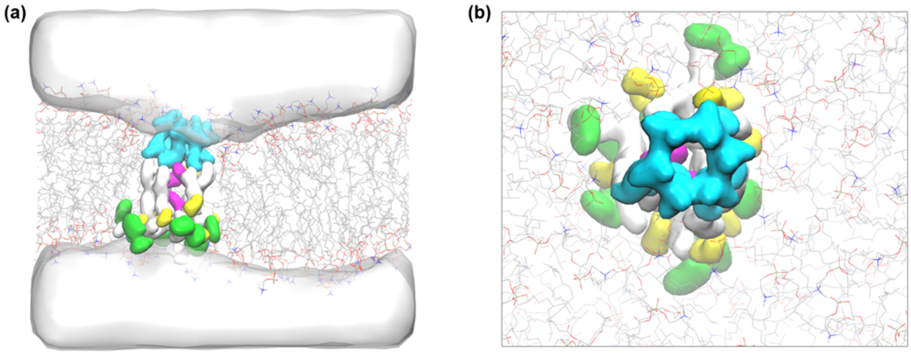

Fig. S24 Structural model of **1** embedded in diPhyPC bilayer. (a) Side view and (b) Top view.

10. Reference

[1]. J. Feng, M. Graf, K. Liu, D. Ovchinnikov, D. Dumcenco, M. Heiranian, V. Nandigana, N. R. Aluru, A. Kis, A. Radenovic, *Nature*. 2016, **536**, 197-200.

[2]. P. Xin, L. Xu, W. Dong. L. Mao, J. Guo, J. Bi, S. Zhang, Y. Pei, C.-P. Chen, *Angew. Chem. Int. Ed*. 2023, **62**, e202217859.

[3]. S. Jo, T. Kim, W. Im, *PLoS ONE.* 2007, **2**, e880.

[4]. B. R. Brooks, C. L. Brooks, A. D. MacKerell, L. Nilsson, R. J. Petrella, B. Roux, Y. Won, G. Archontis, C. Bartels, S. Boresch, *J. Comput. Chem.* 2009, **30**, 1545-1614.

[5]. K. Vanommeslaeghe, E. Hatcher, C. Acharya, S. Kundu, S. Zhong, J. Shim, E. Darian, O. Guvench, P. Lopes, I. Vorobyov, *J. Comput. Chem.* 2010, **31**, 671-690.

[6]. J. C. Phillips, R. Braun, W. Wang, J. Gumbart, E. Tajkhorshid, E. Villa, C. Chipot, R. D. Skeel, L. Kale, K. Schulten, *J. Comput. Chem.* 2005, **26**, 1781-1802.
